# Requirements, Challenges, and Key Components to Improve Onboard Medical Care Using Maritime Telemedicine: Narrative Review

**DOI:** 10.1155/2023/9389286

**Published:** 2023-06-15

**Authors:** Niloofar Mohammadzadeh, Marsa Gholamzadeh

**Affiliations:** ^1^Health Information Management Department, School of Allied Medical Sciences, Tehran University of Medical Sciences, Tehran, Iran; ^2^Medical Informatics, Health Information Management Department, School of Allied Medical Sciences, Tehran University of Medical Sciences, Tehran, Iran

## Abstract

**Introduction:**

Telemedicine has been able to bring healthcare services to all people in far locations such as the sea. Our main objective was to overview the main features, challenges, and requirements of applying telemedicine at sea.

**Methods:**

The electronic search includes all types of papers published in English. It was performed in four databases with keywords to Feb 2023. Next, main categories were defined to extract major concepts. By mapping extracted themes, maritime telemedicine concepts were represented in two conceptual models.

**Results:**

After screening the papers based on title and abstract, 18 articles remained. They can be divided into 13 categories based on their clinical domains. Out of 18 reviewed articles, six articles were published in 2020. The greatest number of studies with five articles was conducted in France. Evidence showed that maritime telemedicine service can be provided to all kinds of ships. Regarding clinical domains, the greatest demand belonged to primary care problems (5 papers) and general health assessment (4 papers). Challenges were divided into four main categories. Moreover, the required services and equipment in four categories were described too. Finally, a conceptual model is represented for providing telemedicine services at sea using satellite Internet.

**Conclusion:**

Despite the existing challenges in providing the required equipment and resources for the implementation of maritime medicine, it has an important role in providing better care for seafarers without time limitations.

## 1. Introduction

All people in the world have the right to obtain a certain standard of health and healthcare in medical emergencies or during their chronic illnesses even in remote areas. One of the remote areas that might not have access to health services for months is the oceans and sea. Ships may be on the water for months and stay away from medical care. Therefore, any health service care can be a life-threatening challenge for sailors and seafarers. Access to healthcare providers always has been one of the most critical issues for offshore crews [1]. According to the International Labor Organization (ILO), access to medical treatments and health services should be guaranteed to seafarers in the same way as for people working ashore [2]. Thus, providing appropriate medical assistance on ships due to disease or accident is one of the most controversial issues in the marine industry.

Nowadays, telemedicine technology has emerged to improve healthcare delivery in remote areas where there is no direct access to healthcare services using telecommunication services. In fact, the origin of telemedicine can be seen in the efforts of sailors to use radio waves to receive medical advice for better care of patients on board [3]. There was no medical care team on the ship with few passengers. Also, specialist medical staff are not always available on cruise ships or navy ships. Consequently, sailors employed different telemedicine-based solutions to deliver medical care advice to their passengers and ship crew. Among them, maritime Telemedical Assistance Services (TMAS) have become more readily available and are considered an integral part of a shipowner's emergency response operations [4]. Equipping ships with satellite Internet service makes remote patient monitoring in the sea easier [5, 6].

Despite the advantages of technology in healthcare, this issue remains neglected, and few studies investigated the employed telemedicine-based solutions in maritime telemedicine in the form of narrative review. None of them were devoted to providing an overview of the characteristics of maritime telemedicine, its benefits, prerequisites, and challenges. This point of view can help health professionals make strategic decisions about applying these technologies in the maritime field more efficacy. Our narrative review is aimed at identifying the main features, challenges, and requirements of applying telemedicine at sea and introducing them.

## 2. History of Maritime Telemedicine

Before the advent of the radiotelegraph by Guglielmo Marconi in 1897, ships had no access to any medical services or the ability to communicate with medical health services from land. With the emergence of radio stations, ships turned to using telecommunication services to receive medical advice via radio [7]. Telemedicine is known as applying telecommunication technology to provide medical care for patients remotely [8]. However, the low bandwidth of radio waves made health information exchange so difficult. Though, using radio frequency technology or voice-only communication to provide telecommunications health services can be an example of telemedicine [9–11].

Applying telemedicine in maritime medicine dates back to the 1930s. The hospital-based radio medical advice center (RMA) was one of the first telemedicine programs established in 1931 by the Cuxhaven Medical Center [12]. Hence, via INMARSAT-ISDN or Iridium-Satellite-Transmission, maritime crews are connected to telecommunication services and expert physicians who have practical experience in maritime routine and emergency medicine [13]. Since the 2000s, modern technologies like telemedicine service can provide a high level of care to passengers and ship crew in medical emergencies. Accordingly, the German Navy built up a telemedicine network that today is installed in all ships and provides a connection to the medical infrastructure ashore.

Because life on board is limited, the transmission of infectious respiratory disease is easier, and controlling it is so difficult. In 2009, more than 2,000 crew members were reportedly infected due to the H1N1 influenza outbreak [14]. Due to the high infectivity of the coronavirus, the COVID-19 epidemic has affected both land and sea. Because infected people are not allowed to disembark in some countries from ships, management of suspected patients has been made more difficult for medical personnel on board [6, 15]. Regarding better management of COVID-19 disease, French TMAS has witnessed a sudden surge in maritime medical consultations compared with the same month in the previous year [16]. It showed that telemedicine consultation can manage patients remotely to control the epidemic concerning isolating rules during pandemics. Furthermore, it has the potential for substantial cost savings within the military healthcare system along with intangible benefits that sustain the military healthcare system downstream.

## 3. Methods

The electronic search includes all types of papers published in English languages, regardless of the publication year, up to the end of Feb 2023. Science Direct, Google Scholar, Scopus, and PubMed databases were searched with predefined keywords regarding applying telemedicine in the sea. The search strategy in the PubMed database is shown in Table 1 as an example. To find relevant studies, the references of selected articles were investigated.

### 3.1. Inclusion and Exclusion Criteria

Articles were included if (1) written in English, (2) study designs included all types of study designs, and (3) focused on using telemedicine-based solutions for patient monitoring at sea or on watercraft. Reasons for exclusion were irrelevance to the topic and articles written in a language other than English reviews rather than original research on this topic.

### 3.2. Screening Process

**Figure 1:**
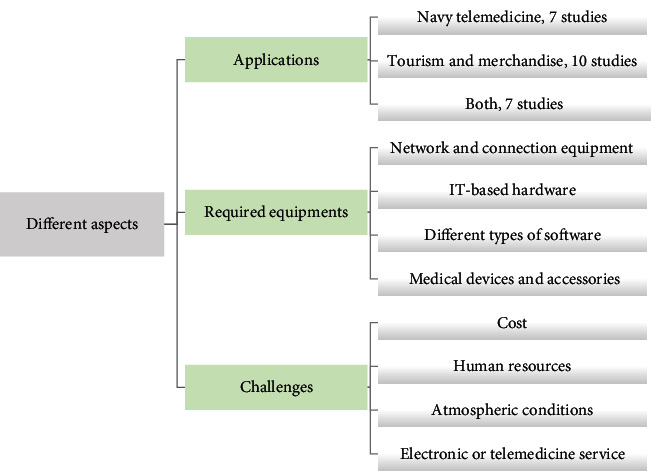
The categories defined in this study.

After the citation was retrieved, duplicate studies were removed. First, the title and abstract of retrieved studies were screened to identify applied solutions about applying telemedicine in the sea or ocean to improve the health of sailors and seafarers. After that, the full texts were screened based on predefined categories to represent the outcomes. No comparative data were found; most published papers were case studies and case series. For this reason, the main subjects were defined to extract major concepts from the articles in the screening full-text phase. These categories are shown in Figure 1.

## 4. Results

A total of 234 citations were found. Among them, 87 articles were duplicates. Thus, a total of 147 studies were screened using the title or abstracts, and 34 articles were fully reviewed and discussed in this study. After screening the papers based on title and abstract, the relevant papers were limited to 18 articles. The selected papers were published in 11 different journals. Six of 18 papers were published by the International Maritime Health journal.

**Figure 2:**
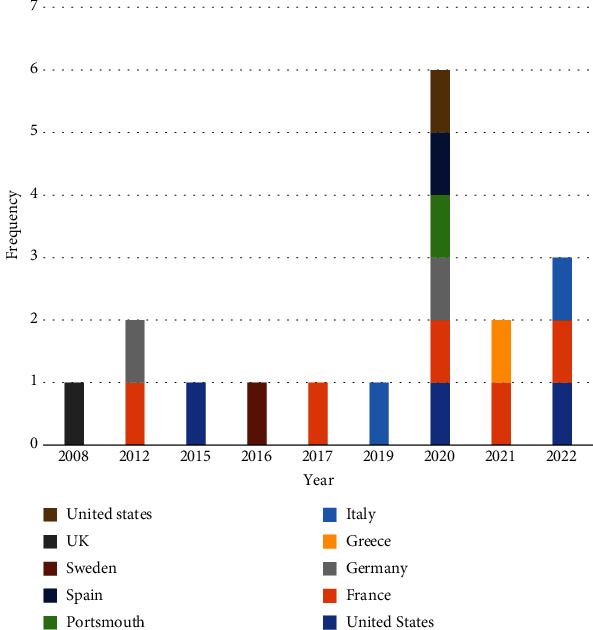
The distribution of articles based on countries and year.

The final articles according to the publication year and country are shown in Figure 2. As we can see, out of 18 reviewed articles, six articles were published in 2020 by different countries [4, 17–24], two studies in 2021 [14, 16, 25, 26], and three studies in 2022 [27–30]. The remained articles were published from 2004 to 2019 [31–39]. Among the countries, France has the greatest number of studies with five articles.

**Figure 3:**
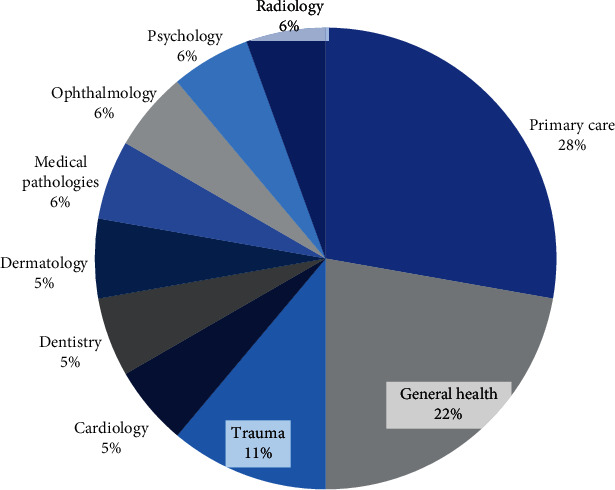
The distribution of articles based on disease and medical domains.

According to reviewed studies, we can classify all reviewed studies into 10 categories based on their clinical domains. Clinical domains with their frequency are described in Figure 3. From the chart, it is obvious that the greatest demand belonged to primary care problems (5 papers) and general health assessment (4 papers). This analysis can be useful to determine the gaps in the literature in terms of maritime telemedicine.

After reviewing the published article, issues raised in the research were divided into several main categories for better comprehension. Although digestive problems are very common at sea, no system has been implemented in this field.

### 4.1. Different Kinds of Ships

All studies were divided into two categories in terms of ship types, military ships with ten studies (37.4%) and nonmilitary ships (commercial and cruise ships) with ten studies (37.4%). Some solutions were implemented for both types (seven studies).

#### 4.1.1. Navy Telemedicine

**Figure 4:**
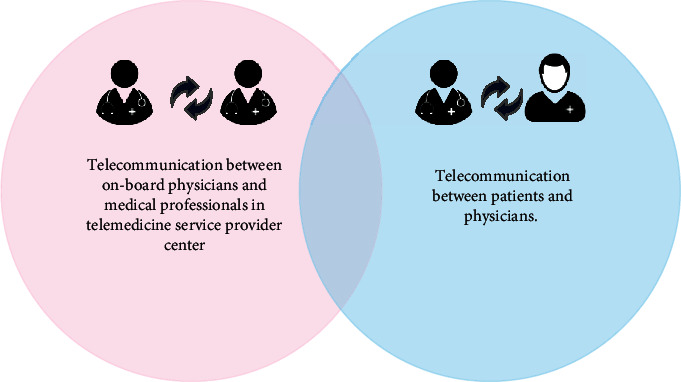
Two types of telemedicine service in the navy context.

Typically, naval ships and submarines have to be stationed for long periods in remote locations at sea and in the oceans to carry out their missions [40]. In a medical emergency, the lack of access to medical facilities is a major concern because the ship cannot stop its mission. Telemedicine is one of the best available solutions to meet the medical care needs of personnel on ships or submarines [22, 40, 41]. In navy telemedicine, there are two kinds of telemedicine services generally that are shown in Figure 4.

*(1) First Type*. The first type of maritime telemedicine refers to the remote communication between the ship's medical staff and the specialist in the telemedicine service center, which has a long history in Spain. The TMAS center of the Spanish Navy offers 24/7 remote monitoring services to seafarers. Specialists in most medical fields are available at the Central Defense Hospital “Gómez Ulla” in Madrid to support military personnel [3, 21]. In addition to teleconsultation and video conferencing, this telemedicine service provides some additional services such as teleradiology, telecardiology, teleophthalmology, teleotolaryngology, teledermatology, and teleultrasound and the transmission of vital signs like electrocardiography (ECG) in a real-time mode [21].

*(2) Regarding the Second Type*. This type of service refers to remote communication between the medical specialist in the medical center and the patients on the ship to follow up on their health status. It is more specific to ship personnel and their periodic health assessment [13].

#### 4.1.2. Cruise Ship Medical Tourism alongside Merchandise Ships

Cruise vacations have become more popular every year for the past two decades. During the 2008s, the maritime tourism industry estimated that it had 13.05 million cruise passengers worldwide [42]. The cruise industry is a special part of the tourism industry. Today, in addition to the welfare and entertainment services that this industry provides to passengers, they are looking for new ways to provide more health services on ships alongside the medical tourism industry [43]. Due to the high cost of medical care, these costs are not included in the fare on cruise ships. But today, most cruise ships can use telemedicine services using existing advanced equipment. The use of telemedicine services can support onboard medical staff and provide them with better management of medical events on ships [2].

*(1) Medical Staff Problems*. Cruise ships are like floating cities. They offer limited professional medical services through licensed (international or domestic) physicians and nurses. Under international law, only vessels with 100 or more crew members on an international voyage of three days or more required a specialist doctor on board [18, 20, 44]. Konrad et al.'s study on ten cruise ships found that 1,627 therapeutic events were recorded in just six months [44]. However, there is a medical ward inside a pleasure boat or cruise ship. But they can provide limited professional medical services by trained personnel and internationally licensed physicians. So, one of the biggest problems with cruise ships regarding medical staff is that the hired medical personnel are usually not specialists in various fields of medicine.

*(2) Applied Solution*. If a passenger or crew member becomes ill or is injured, telemedicine could be beneficial for remote patient monitoring and providing essential medical care [16, 17]. Since cruise ships are quite equipped with high-speed satellite networks and advanced equipment, telemedicine programs can provide teleconsultation for onboard medical staff for getting in touch with telephysicians who are not physically present [45]. One effective telemedicine-based program that has been successfully implemented for merchant and commercial vessels was called MEDASHIP. It was an interactive telemedical consultation to improve onboard medical care for passengers and crew members [46]. In this context, Henes et al. implemented a secure and stable method of image and radiology report transmission between an onboard hospital and a land-based radiology department to support ship medical staff with expert consultation [20].

*(3) Future of Telehealth in Tourist Ships*. Likewise, the Cleveland Clinic believed that telemedicine could be considered an incredible opportunity to provide telemedicine services in remote areas [47, 48]. Cruise ships are equipped with all required facilities for high speed and a secure connection line for telehealth implementation such as expensive satellite communication and onboard medical devices for providing software-based videoconferencing for a ship at sea [44]. Researchers believe that the future of telemedicine services on cruise ships is very bright, which could provide an intelligent ecosystem to better support the health of the crew and passengers [44].

*(4) Merchandise Ship*. Workers on commercial and fishing boats may also need medical advice due to accidents, poisoning, or even underlying illness. Thus, any kind of teleconsultation service could be beneficial [38].

### 4.2. Different Types of Telehealth and Telemedicine Services

In the reviewed articles, different types of telehealth and telemedicine services were used to communicate from the ship to medical centers. The investigation showed that all these methods can be divided into nine categories. Of 18 studies, 7 (38.89%) articles devoted to teleconsultation, four (22.22%) articles were conducted for televisit, two (11.11%) articles were related to teleradiology, one (5.56%) study devoted to teledermatology, one (5.56%) article devoted to teledentistry, one (5.56%) article devoted to telecardiology, one (5.56%) article devoted to teleophthalmology, and one (5.56%) article was done regarding telepsychiatry. As it is apparent, teleconsultation and televisit are the most frequent methods in maritime telemedicine.

Telemedicine technologies can be broadly divided into three types including synchronous telemedicine, asynchronous or store-and-forward telemedicine, and remote patient monitoring. Based on this, among the 18 reviewed articles, 61.11% used store-and-forward model for communication, 33.33% of services employed synchronous telemedicine, and one article used remote patient monitoring. Among the services applied store-and-forward model, most of them used email services to send patient information.

To use telemedicine services at sea, they used high-speed Internet or satellite Internet to send information. Among the literature, 17 out of 18 studies use satellite Internet. Though 4G- and 5G-like satellite Internet is wireless, 4G/5G is not completely wireless and it should hook up to a fiber Internet source. Ships are dependent on satellite communication when out of range of coastal systems. A wide variety of satellite communication systems are available. Inmarsat systems C, Fleet 77 (and probably BGAN soon) are the only systems to satisfy IMO GMDSS (Global Maritime Distress and Safety System) requirements for satellites. However, these do not cover polar regions. Only iridium covers the full globe, including the Arctic, but with limited bandwidth [49]. Hence, satellites are the key technology that allows instruments to communicate from anywhere on the ocean surface. However, in reviewed articles, they did not mention the communication details that they used. One study applied a virtual private network (VPN) alongside a satellite connection service to secure data on the infrastructure from outside. A VPN has the ability to create a secure and encrypted connection over less secure networks.

### 4.3. Software and Hardware

Although there are not many explanations about the software and hardware of the systems used in the articles, a summary of their characteristics is given in Table 2 based on type of telemedicine service.

### 4.4. Required Equipment and Services

Studies have shown that some equipment and services are needed to establish telecommunication in maritime telemedicine. In each type of services used in marine telemedicine, different equipment has been used according to the purpose of the study. The equipment by type of telemedicine and communication service is listed in Table 3.

Although in most of the articles the applied equipment is not clearly mentioned, the equipment can be generally divided into four categories, (1) network and broadcast connection equipment, (2) IT-based hardware prerequisites, (3) different types of software, and (4) medical equipment, devices, and accessories.

#### 4.4.1. Network Availability and Connectivity

More than two-thirds of the earth's surface is covered by water. So, for worldwide Internet access, there are cables even under the sea for point-to-point connections [50]. But these connections cannot provide access to the Internet at sea level [51]. Thus, providing sufficient bandwidth and high-speed Internet access to transmit a high range of data is the main concern to offer a telehealth service at sea and ocean. Nowadays, most of the ships sailing at sea are equipped with satellite or radio Internet. The advent of such services provides real-time access and data transformation from ships to hospitals or healthcare service providers for seafarers [5]. With these new technologies, medical professionals could offer a level of medical assistance in the most remote locations even in unstable weather conditions. Such communication services need ground equipment, a satellite amplifier to change the incoming signal, and a satellite to receive and transmit the signal to receive a signal from the earth and transmit an incoming signal back to earth [52]. Grounded equipment for the satellite network can be placed on ships [53]. In most published articles regarding maritime telemedicine, satellite technology is the most optimal service for using telemedicine service at sea to establish a reliable connection.

#### 4.4.2. IT-Based Hardware

The most essential means of communication between a service provider and a ship's crew is a computer, laptop, or tablet. In addition, a server is needed to store information on the server side [54]. Most of the time, the physician needs to talk to the patient, examine he/her, and give the necessary instructions to the resident doctor on board. Thus, real-time video and audio content is one of the main prerequisites for launching telemedicine at sea, so equipping with a connected video camera or webcam is essential for effective communication through teleconsultation [55].

#### 4.4.3. Software Services

In any telemedicine service, the desired system is designed at different levels of accessibility. To communicate properly with an onboard physician, a subsystem for the service provider and a subsystem for the service receiver must be planned [12, 20, 40]. In addition, there is usually a subsystem at the admin level to manage the admin level to improve security and system management [56]. Marine telemedicine systems also have all of these components, regardless of the unique characteristics of each system. It should be noted that all these subsystems are interacting with each other constantly [4, 32]. Additionally, some studies referred to developing a personal mobile-based application for personal health monitoring in the context of telehealth service [13].

#### 4.4.4. Exclusive Medical Equipment and Devices

Unpredictable accidents and illnesses on ships moving on the water can lead to a medical emergency. Additionally, evaluating seafarers' continuous wellness is another important issue. Getting help from telemedicine services is not only possible by providing high bandwidth [57]. But for the proper management of patients, first of all, equipment is needed to measure vital signs and accurately send these results to the telephysician. The five vital signs are temperature, blood pressure, blood sugar, SpO_2_, and ECG. However, sometimes doing blood tests or performing medical imaging is essential to determine if a patient suffering from a serious situation such as dengue fever, heart attack, or bone fracture [13, 16, 20]. Some of this equipment applied in different studies include a wireless pulse oximeter, electrocardiogram (ECG), telespirometry, teleultrasound, and mobile digital radiography unit. Another type of equipment used in this field is concerned with smart medical kits which consist of combining digital and medical diagnostic devices such as quick tests with a smart connection application on a tablet or personal computer that can be kept on board ships [41].

### 4.5. Challenges and Their Applied Solutions

Telemedicine service provides a physician virtually on board, but providing secure and real-time telehealth service for maritime has encountered some challenges. All of these challenges can be divided into three categories, cost, human resources, and atmospheric conditions. These challenges mentioned in various studies are listed in Table 4.

#### 4.5.1. For Cost

To reduce the costs associated with providing telemedicine services, this type of service is considered a mandatory service along with other services provided to passengers. On military ships, on the other hand, the directors of naval establishments are trying to cover these services with insurance [12].

#### 4.5.2. For Human Resources

Trained personnel are required to use telemedicine services and communicate with provider centers. As a result, to protect the health of ship passengers, courses for medical personnel stationed on ships are held by telemedicine service companies [3].

#### 4.5.3. For Atmospheric Conditions

Unfavorable and unpredictable weather is one of the problems in communication between ships and telemedicine service providers. In addition to the impact of weather problems on Internet communications, this problem can affect the accuracy of medical equipment on the ship. To overcome these problems, they usually use satellite Internet and fix medical equipment and kits.

#### 4.5.4. For Telehealth Service

Usually, telemedicine involves the circulation of highly sensitive information such as personal information or patient health information. Therefore, to ensure the confidentiality and security of patients' information, it is necessary to record and maintain all conversations and information transfers between the physician residing in medicine (or the patient receiving the service) and the specialist physician [58]. In some studies, to authenticate patient records, tokens were used [33].

Today, the latest innovations in medical technology using artificial intelligence help healthcare providers provide more efficient services by equipping patients with wearables and other tools for remote patient monitoring. The Internet of Things consists of connected devices and sensors that transmit a constant stream of data to the supervising physician for better patient monitoring. These tools provide health managers with better, more accurate, real-time access to data and improve decision-making. The use of these kinds of tools in marine telemedicine has not yet been widespread, due to the lack of infrastructure, high costs, and the need for equipment.

The most important problem mentioned by all the articles was the long time it took to get to the beach to get medical services. Other benefits of using telemedicine compared to routine medical care at sea are listed in Table 5.

## 5. Discussion

This study provides an overview of maritime telemedicine through a narrative review to map the key concepts focusing on the key features and main challenges in a rapid form of knowledge synthesis. By applying these kinds of solutions, the main concept and the characteristics of this type of telemedicine service were investigated by defining some categories to overview their characteristics and perquisites [59]. Our review revealed that telemedicine has a critical role to provide better care for seafarers without no time and place restrictions. Examining the different applications of maritime medicine at sea shows that this type of service can help seafarers in emergencies as well as provide routine medical services to passengers and mariners.

**Figure 5:**
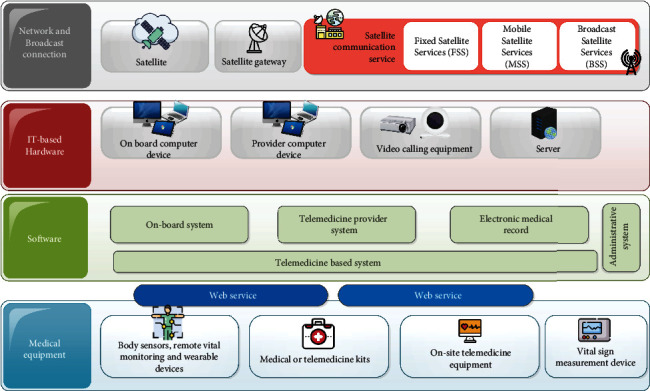
Different types of required equipment and suggested architecture.

The implementation of telemedicine service requires the infrastructure and equipment that were represented in the findings section. According to literature review, the suggested platform based on required equipment is illustrated in Figure 5 as general architecture.

Despite the many benefits of using telemedicine in the maritime industry, there are still some challenges that can face telemedicine services with failure. In the results section, we organized these challenges into four main categories and subcategories. Due to the frequent use of telemedicine services in recent years, we provided some recommendations regarding some of the challenges in each category.

As explained, telemedicine service is one of the services provided to passengers, even on cruise ships, merchanting ships, or even on military ships to provide better care for seafarers. Thus, providing seafarers with telemedicine service can fulfill the right of everyone regarding equal access to health services in a short time [2]. Although air ambulances are used in many cases to provide medical services to seafarers, according to Horneland [60], this type of service also faces challenges due to bad weather conditions and long service delivery time.

**Figure 6:**
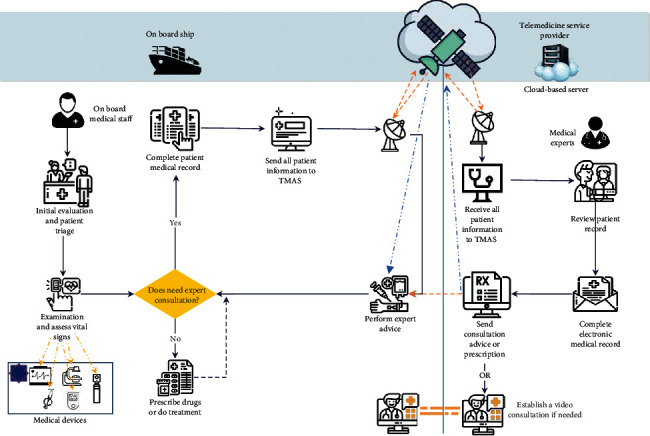
The general model of maritime telemedicine.

After analyzing the working environments, this study designed an infrastructure consisting of a network, medical equipment, and server required for maritime telemedicine. Based on the findings, all equipment and prerequisites can be summarized on a general model presented in Figure 6 using satellite Internet. This type of service provides a reliable, high-speed connection to facilitate remote medical services for vessels traveling on water. However, access to high-speed Internet services has been very difficult for seafarers in the past. To establish telemedicine, appropriate hardware infrastructure must be established inside the service provider's facility and onboard ships. This infrastructure must prevent the breach of personal information and security when using web-based systems and remote video consultation, and all equipment must be reliable and secure. Considering the advantages of wireless sensors and IoT devices in transmitting health data, vital sign monitoring sensors are also considered in this model. In this way, the measured data could be transmitted to the main telemedicine service using an integrated gateway via connected Bluetooth devices. The data storage server is located in the center of the telemedicine service provider. In this type of service, cloud-based services can enable doctors to remotely access patient data. However, when using cloud services, it is necessary to pay attention to security issues.

Although the investigations showed the advantages of applying telemedicine services in different medical conditions, implementation of such services needs high-cost equipment and infrastructure. Electrocardiography (ECG) devices to provide telecardiology services [31], portable imaging equipment for teleradiology [20], point-of-care ultrasound [18], and dental units connected to the satellite network to provide teledentistry services are some examples which needed to implement telemedicine in sea [17]. As the results showed, part of the studies in maritime telemedicine was devoted to the general health assessment of employees and periodic follow-up. Therefore, the development of an electronic health record platform for seafarers, in addition to making it easier for them to provide remote medical services, is also considered one of the prerequisites in the design of this type of service [33].

Although the sea and navy are far from land, they are not safe from a pandemic or transmission of infectious diseases. Among reviewed articles, 12% of them were devoted to controlling pandemic diseases like Ebola [35] and COVID-19 [14, 19, 23].

In similar studies, teleconsultation advice analysis provided by medical specialists to medical staff in ships from October 2012 to the end of 2018 showed that infectious diseases were the most common cause of teleconsultation [4]. On the other hand, from the beginning of the epidemic, using telemedicine-based services became crucial. Timely action and remote patient care using AI service can save many lives before they reach the medical center [61]. Providing critical care for infected patients who suffer from severe types of COVID-19 seems essential [62]. However, in many cases, passengers may not be allowed to leave the ship, even in the event of an emergency due to a pandemic onboard [15]. In such cases, receiving medical advice remotely, video consultation, or monitoring symptoms via wireless sensors were employed [6, 16, 19].

In other kinds of telemedicine services, the combination of cloud computing, body area network, IoT devices, big data analysis, and telemedicine technology services has provided new opportunities for more efficient data transformation, efficient data storage, and safe exchanging of health data [63–65]. As a result, telemedicine technology can use real-time data and IT devices to enable higher quality telehealthcare. In this type of technology, a wearable sensor placed on the body is connected to an edge node in the IoT cloud, where data is processed and analyzed to define health status. By solving the problem of high-speed Internet, this type of technology can also be used in marine telemedicine [66]. Considering the positive effects of cloud computing and the need for a platform for storing and exchanging data, it seems that this type of service in the marine telemedicine platform should be given more attention.

This study encountered some limitations. Only papers published in the English language were included. However, the gray literature, which may provide valuable information to researchers, was also examined in this study. Although this study is not a comprehensive study, these findings can provide researchers with an overview of the characteristics of maritime telemedicine services.

## 6. Conclusion

Despite increasing the number of ships and seafarers, direct access to medical care is an obstacle in maritime working conditions. Evidence showed that though the treatment of patients in isolated and remote places is not easy, seafarers can spend time on ships safely and more conveniently by integrating medical care and technology through telemedicine service. We investigated some challenges that staff faced with them like lack of healthcare personnel, lack of telecommunication services, high cost of equipment, absence of telemedicine equipment, and the distance from the main medical center in our review. Consequently, we proposed the conceptual framework to develop telemedicine-based services for all kinds of ships to access telemedicine services. The proposed framework provides new insight into the best practices for health informaticians according to practical applications of maritime telemedicine.

## Figures and Tables

**Table 1:** Keywords and search strategies applied in this study.

Search strategy
1. (“marine”[Title/Abstract]) OR (“navy”[Title/Abstract]) OR (“sea”[Title/Abstract]) OR (“seafarer”[Title/Abstract]) OR (“sailor”) OR (“maritime”) OR (“Cruise Ship”[Title/Abstract]) OR (“Merchant shipping”[Title/Abstract]) OR (“commercial shipping”[Title/Abstract])
2. (“Telemedicine”[Mesh]) OR (“Telemedicine”[Title/Abstract]) OR (“Telehealth”[Title/Abstract]) OR (“e-health”[Title/Abstract]) OR (“Mobile health”[Title/Abstract]) OR (“mhealth”[Title/Abstract]) OR (“m-health”[Title/Abstract]) OR (“ehealth”[Title/Abstract]) OR (“digital health”[Title/Abstract])
3. 1 and 2

**Table 2:** Description of hardware and software applied in maritime telemedicine.

	Software	Hardware
Teleconsultation	eMass digital platform CIS system AppliCCMM software French TMAS Swedish TMAS Linux operating system Radio medical advice system	Portable telemedicine equipment collecting ECG, blood pressure, and oximetry signals USB devices

Telecardiology	AED Plus software	Automated external defibrillators (AEDs) with telemedical capacities on board merchant ships Telecardia equipment

Teleophthalmology	TMAS service	Digital camera

Teleradiology	Academic maritime medical advisory service PACS Radiology information system	Ultrasound equipment Mobile digital radiography unit

Telepsychiatry	DigiGone®, SecureChat™ system	Digital camera

Televisit	CCMM telehealth services French TMAS Marine Doctor (M Doc) Free open-access software Skype	Photography device PC with 64-bit Windows versions Portable vital monitoring devices

**Table 3:** Employed equipment and communication services.

	Teletype service	Equipment	Communication
Asynchronous/store and forward	Teleconsultation Telecardiology Teleradiology Teledermatology Televisit	A network cable to the ship's satellite communication system and email program Mobile digital radiography unit Telemedicine case Portable onboard units	Sending digital photos via email, a telephone contact Teletransmission of photographs Virtual private network (VPN) via satellite Internet Satellite communication Email service API and asynchronous communication

Real time audio-video	Teledermatology Teleconsultation Telepsychiatry Televisit	High-resolution external camera Secure video chat equipment Digital photography Bluetooth ECG recorders, oximeter with Bluetooth, sphygmomanometer with Bluetooth, 12-lead electrocardiograph with Bluetooth Dermoscopy, Cisco webcam video jabber, Global med cart	Satellite Internet and store-and-forward type Satellite communications to provide a low-bandwidth, encrypted video teleconferencing capability for the maritime industry Teletransmission of photographs with satellite Internet/5 g Real-time service

Interactive audio with store and forward	Teledentistry Teleradiology Televisit Teleophthalmology	Dental cases Digital photography Large radiology cart-based systems Digital radiography unit and PACS Portable and connected ultrasound devices Portable and connected ECG device Wireless and intelligent ophthalmology equipment Laptop-sized machines Handheld ultrasounds that can connect to a tablet or smart phone ECG recorders Slit lamp photograph Audio and mobile phone service Portable onboard units	Telephone-based communication Cloud service Synchronous telephone consultation, store-and-forward e-mail systems Radio medical service

Phone-only, form-based Internet prescribing	Teleconsultation	Audio and mobile phone service	Telephone-based communication

Remote patient monitoring	Teleconsultation	A water-proof personalized USB drive provided to each seafarer	Satellite Internet

**Table 4:** The main challenges and their subdomain.

Main categories	Related challenges
Cost	High equipment costs on board
	High costs of implementing telehealth service in ships for communication with the telemedicine service provider
	Lack of insurance coverage
	Communication via satellite
	High costs of medical equipment such as telespirometry, radiology units, and wireless devices

Human resources	Requires a 24-hour standby person to communicate and receive advice
	Need ship-trained personnel to carry out medical orders and communicate properly with telemedicine services
	The telephysician should know the maritime vocabulary

Atmospheric conditions	Impact of bad weather on quality of bandwidth
	Impact of bad weather on onboard equipment

Telehealth service	Designing an easy-use user interface (UI) and intuitive handling of the system
	Data privacy and confidentiality
	Documentation and data storage
	Lack of data standards for interoperability between maritime systems and telemedicine providers
	Lack of necessary infrastructure for implementation and usage of IoT and big data analysis

**Table 5:** Comparing maritime medical care with common medical care.

	Routine medical care at sea	Using telemedicine service at sea
Patient safety	The patient's life may be endangered due to lack of access to medical services at the right time	With proper counseling and taking primary medical intervention, the patient's life can be saved or lead to an early diagnosis
The need for specialized and trained personnel	Without the possibility of consulting with specialists, there is a need to hire an expert medical staff on board	Employing the telemedicine service and providing the necessary equipment, trained ship staff/general practitioners/nurses can also receive specialized medical services
Insurance	Usually cover insurance	It depends on the ship and the type of insurance contract
Cost of medical service	It can save money by taking timely action and preventing the consequences of an accident or illness	The types of procedures and services that must be provided to the patient may increase due to the lengthening of the patient's processing time which can lead to increased costs

## Data Availability

No underlying data was collected or produced in this study.
